# Surgical Management of Temporomandibular Joint Ankylosis in Ankylosing Spondylitis

**DOI:** 10.1155/2011/854167

**Published:** 2011-03-24

**Authors:** Andrew M. Felstead, Peter J. Revington

**Affiliations:** Department of Oral & Maxillofacial Surgery, Frenchay Hospital, Beckspool Road, Bristol BS16 1LE, UK

## Abstract

Relatively few patients develop such severe degenerative temporomandibular joint (TMJ) disease that they require total joint replacement. Current indications include those conditions involving condylar bone loss such as degenerative (osteoarthritis) or inflammatory joint disease (ankylosing spondylitis, rheumatoid, and psoriatic). Ankylosis of the temporomandibular joint (TMJ) secondary to ankylosing spondylitis remains an under investigated entity. We aim to provide an overview of treatment objectives, surgical procedures, and our experience with total TMJ replacement for this condition.

## 1. Introduction

Prosthetic replacement of the TMJ is a procedure that has undergone a technological revolution over the last decade. Early joint prostheses (e.g., Bowerman-Conroy) developed in the 1970s were functional but large and difficult to use.

New technology has led to the development of systems that are custom fitted, well tolerated, and deliver good clinical outcomes.

## 2. Background

The temporomandibular joint (TMJ) is a diarthrodial atypical synovial joint that is capable of both rotational and translatory movements. The TMJ is formed by the mandibular condyle and the glenoid fossa of the squamous part of the temporal bone and is separated into upper and lower cavities by a fibrocartilagenous articular disc.

Due to the inherent surface structure of the TMJ, it shares the tendency of involvement by ankylosing spondylitis (AS) as with other fibrocartilagenous structures such as the symphysis pubis and the intervertebral discs [1]. 

Although the reported incidence of TMJ involvement in AS is not uncommon, its onset appears to be insidious, and patients with TMJ involvement tend to be older and have more extensive spinal and peripheral joint disease. Involvement of the TMJ appears to give rise to few serious symptoms until pain, stiffness, and gross restriction of jaw movement has occurred [2].

In spite of the high frequency of TMJ involvement, ankylosis secondary to AS seems to seldom ensue, and relatively few patients develop such severe degenerative temporomandibular joint (TMJ) disease that they require total joint replacement. This is reflected in the paucity of literature on the topic, with only approximately 11 cases reported worldwide.

However, when ankylosis occurs, it is an extremely disabling affliction that causes problems with mastication, digestion, speech, appearance, and access to routine dentistry. It also has an impact on the psychological development of the patient with concerns related to an inability to open the mouth [3].

 Patients typically present with increasing pain on eating, often associated with progressive limitation of mouth opening accompanied by radiographic evidence of joint degeneration (loss of joint space, osteophytes, surface erosion, and ankylosis). Clinical indications for intervention involve a combination of factors: restricted mouth opening (<35 mm), dietary scores (liquid scores 0, full-diet scores 10), occlusal collapse (anterior open bite or retrusion), excessive condylar resorption and loss of vertical ramus height, pain score >5 out of 10 on a visual analogue scale, and other quality of life issues [4].

## 3. Treatment Objectives

Removal of the ankylotic mass, restoring the form and function of the joint, mouth opening, relief of upper airway obstruction, and prevention of recurrence are of utmost importance. Facial remodelling and restoration of occlusion are also significant aims which follow on from the above.

Meticulous preoperative planning, perioperative management, and diligent postoperative care remain the keys to providing successful surgery [5]. 

**Figure 1:**
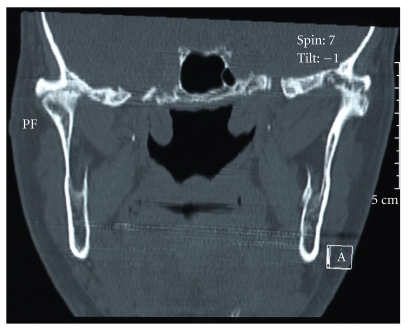
Coronal CT scan showing bilateral TMJ ankylosis in ankylosing spondylitis.

Preoperative imaging by computed tomography of the ankylosed joint in sagittal, coronal, and axial planes is mandatory (Figure 1). This is particularly useful in assessing the extent of the bone mass medially. When used with contrast, proximity to the important vascular structures in the infratemporal fossa can be assessed.

Administration of anaesthesia to patients with condylar ankylosis poses specific problems and demands expertise. Laryngoscopy and patient positioning can be difficult in patients with minimal interincisal opening and fixed flexion deformity of the cervical spine. Specific anaesthetic challenges arise in relation to intubation and securing the airway, awake extubation, and postoperative maintenance of a patent airway. Options for securing the airway include [5]

- nasotracheal intubation via direct laryngoscopy (compromised but adequate mouth opening),
- fibreoptically assisted nasotracheal intubation,
- blind nasal intubation,
- elective tracheostomy under local anaesthesia.

In practice, patients often present with near total trismus and altered upper airway anatomy, thus providing inadequate mouth opening for direct laryngoscopy. An awake tracheostomy is technically demanding owing to patient neck position and should be considered only as a last resort. 

## 4. Surgical Correction of Ankylosis

The basic principle in surgical release of ankylosis is the aggressive resection of the ankylotic mass. This technique is broadly termed gap arthroplasty and frequently employs the use of an interpositioning material to prevent recurrence.

Gap arthroplasty was first described by Abbe in 1880 [6]. In this procedure, a block of bone is removed, either the complete condyle or a full-thickness section of bone leaving a gap between the ascending ramus and the temporal bone. The width and extent of bone removal is crucial, and a minimum distance of 1 cm is necessary to prevent reankylosis [7]. Complications are common, and the operation may also involve ipsilateral and/or contralateral coronoidectomy to achieve satisfactory maximal interincisal mouth opening. 

Reankylosis is of course a major concern and therefore interpositioning of autograft or allograft is thought to deter this. Essentially the same procedure is followed, but the created gap is obliterated by the interpositional material. A variety of grafts have been used for lining the joint space after resection of the ankylotic mass. Using autogenous temporalis muscle as a myofascial flap has gained popularity due to its proximity to the operative site. Other graft materials have included fascia lata, masseter muscle, and auricular cartilage [8]. Alloplastic interpositional grafts have included materials such as silastic, proplast/Teflon, metallic fossa implants, and acrylic marbles [9]; however, they have met with more failure than success.

Functional restoration can be achieved by the procedures outlined above, yet neither of them attempts to reconstruct the resected joint. Reconstruction of the condyle is important to prevent an open bite, establish posterior face height, and avoid pseudoarticulation that may promote reankylosis [5]. It is here that attention is turned to temporomandibular joint replacement.

Many techniques have been described and include both autogenous (fibula, metatarsal, clavicle, ileac rest, sternoclavicular, and costochondral) and alloplastic (acrylic, compressible silicone rubber and total joint systems) options [10, 11].

The most widely accepted autogenous reconstruction is the costochondral rib graft. However, in patients with arthropathy, there are a number of potential problems. The need for long-term steroids may weaken the graft, and there is the possibility of further ankylosis affecting the reconstructed joint [12]. In addition, there is the potential for donor site morbidity [13] including thoracic wall deformities and variable behaviour of the graft.

Drawing parallels from orthopaedic surgery and advocates of alloplastic reconstruction opposes placing an autogenous tissue, such as bone, into an area where reactive or heterotopic bone has formed at least once [5]. Therefore, in patients with ankylosing spondylitis, the total replacement of the TMJ with an alloplastic joint system has become the treatment of choice. These allow closer reproduction of the natural anatomy, avoids donor site morbidity, decreases the risk of reankylosis, and reduces operation time. Furthermore, they allow for immediate physiotherapy and rehabilitation with consequent increased benefit to the patient.

In the United Kingdom, there are three prostheses on the current market.

The Christensen system consists of a fossa and condyle prosthesis both made from cobalt-chrome alloy and is the system subject to the longest period of followup.

TMJ concepts (formerly Techmedica) have up to 17 years followup with 90% success rates [14]. These are computer-aided design/computer-aided manufacture (CAD/CAM) custom-made prostheses constructed on a stereolithographic model following a 3D CT scan. The glenoid fossa component is constructed from titanium mesh bonded to the articular surface of high-molecular-weight polyethylene. The condylar component head is cobalt-chrome alloy, and the remainder of the body is titanium and is secured to the ramus of the mandible by screws.

Lorenz makes a stock prosthesis with similar components to the TMJ concepts prosthesis.

Whichever operative strategy is employed, it is important to note that these techniques all rely on aggressive postoperative physiotherapy to maintain optimal results. 

**Figure 2:**
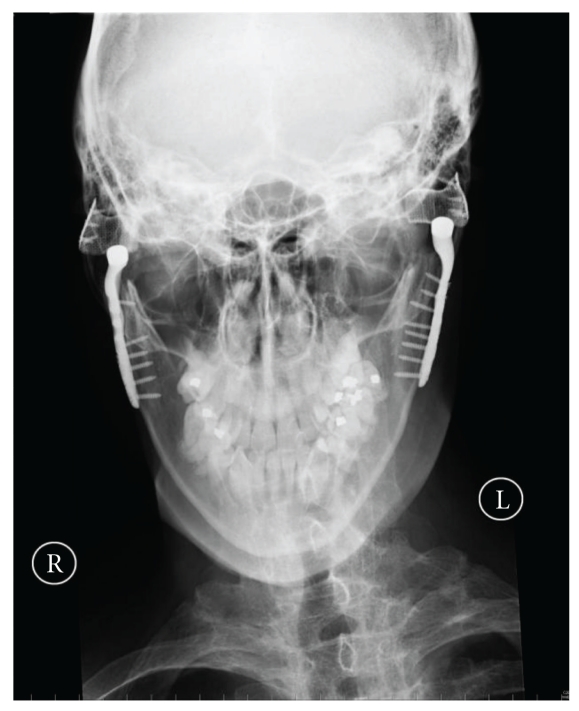
Postoperative PA mandible radiograph showing TMJ concepts prostheses in situ.

**Figure 3:**
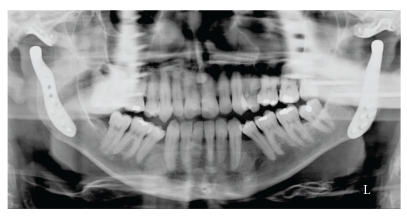
Postoperative OPT radiograph showing TMJ concepts prostheses in situ.

Since 2006, we have performed 19 total prosthetic joint replacements using the TMJ concepts system on 18 patients. Two patients presented with ankylosis, one of whom had ankylosing spondylitis affecting both joints [15] (Figures 1, 2, and 3). The result of our overall experience with the TMJ concepts system is reflected in Table 1. 

## 5. Discussion

Ankylosing spondylitis (AS) is a chronic, usually progressive inflammatory disease of the axial skeleton often affecting the spine and sacroiliac joints. The reported prevalence of AS is 1% and appears to be similar for both Caucasian and Chinese populations [16, 17]. Peripheral joint involvement occurs in 10–30% of patients and may occur in an irregular fashion [18].

It is known that ankylosing spondylitis involves fibrocartilagenous structures such as the symphysis pubis and the intervertebral discs, the hallmark being enthesopathy with a tendency to bony ankylosis in the affected joint. 

 The involvement of the temporomandibular joint (TMJ) in ankylosing spondylitis has not been widely recognized, and its incidence is disputed. Former studies have reported the involvement of the TMJ in AS between 4% and 32%. However, these studies were either only radiological [19] or used only insufficient clinical [20] or radiological [2] examination methods. Most investigations depended on the orthopantomogram which gives unreliable data on the TMJ [21]. In a combined clinical and radiographic study, fifty AS patients were examined. Ten patients showed radiological evidence of AS; however, only one was judged to have AS on clinical grounds [21]. This disparity in clinical and radiological signs suggests that the TMJ rarely seems to be a severe problem for AS patients. It is felt that most TMJ symptoms may be secondary to muscle spasm, occlusal factors, and postural imbalance [18], and the literature is only sparsely populated with cases of true ankylosis. Nevertheless, routine clinical examination should be performed to detect the development of this rare but important condition. 

It should be emphasised that TMJ replacement for this patient group presents with many difficulties, both in terms of the anaesthetic challenges outlined above and potential surgical pitfalls. Approach to the joint is often through distorted anatomy. The risk of complications includes scar formation, facial nerve damage, gustatory sweating, and external auditory meatus perforations, and the most dreaded of note are perforation into the middle cranial fossa and severe bleeding from the medial infratemporal fossa. It remains to be seen what the long-term outcomes of joint prostheses will be and, as yet consensus is therefore lacking on the optimal timing of surgery. TMJ concepts prostheses utilise materials well proven in orthopaedic reconstruction of the knee and hip. Although life expectancy of these prostheses is unknown, studies have shown service life of 8 years [22] and 10 years [14] without evidence of untoward wear or failure. It is postulated that due to the TMJ being a non-load-bearing joint, these devices may have a minimum lifespan of 15 to 25 years; however, in patients with a lifetime chronic illness, it remains difficult to counsel them to early or late intervention in respect of any potential need for further joint replacement. 

In the UK, TMJ replacement surgery is restricted to Oral and Maxillofacial Units with a special interest and referral guidelines for this expensive and technique-sensitive procedure are stringent. A national database is currently in production for analysis of outcomes [4], which along with data from other units worldwide will provide evidence for future recommendations.

Potential disadvantages with alloplastic TMJ reconstruction relate mainly to wear at the articular surface. Metal-on-metal prostheses (Christensen) have been documented as causing foreign body giant cell reactions [23], whilst all patients receiving Cobalt-chrome prostheses should be allergy tested against potential hypersensitivity reactions. 

The results from our own experience are encouraging. Our only case of ankylosing spondylitis improved maximal interincisal opening from 10 mm to 30 mm, whist the case series overall improved the maximum interincisal opening on average by 123%. Outcomes have also shown an improvement in pain scores and weight gain; however, data was not available on all patients and is the subject of a further study.

## 6. Conclusion

We strongly believe that restoring function of the mandible in TMJ ankylosis is of utmost importance. TMJ replacement surgery is low volume with relatively few surgeons carrying out this complex procedure. National guidelines for TMJ replacement have been established in the UK.

Ankylosis of the TMJ is a challenging problem for both the patient and surgeon. Over the years, fundamental principles of TMJ surgery and “trial and error” have shaped the evolution of techniques now used to correct the problem. With the advent of newer technology, adult patients with severe TMJ ankylosis have the option of complete TMJ prosthetic rehabilitation.

## Figures and Tables

**Table 1:** Summary of results for maximal interincisal opening (MIO) using TMJ concepts total temporomandibular joint prostheses.

Number of patients	Male	Female	Right TMJ replacement	Left TMJ replacement	Bilateral TMJ replacement	Mean pre-op MIO (mm)	Mean post-op MIO (mm)	Mean improvement in MIO (mm)
18	2	16	10	8	2	13	30	17

## References

[1] Chow TK, Ng WL, Tam CK, Kung N (1997). Bilateral ankylosis of temporomandibular joint secondary to ankylosing spondylitis in a male Chinese. *Scandinavian Journal of Rheumatology*.

[2] Davidson C, Wojtulewski JA, Bacon PA, Winstock D (1975). Temporo mandibular joint disease in ankylosing spondylitis. *Annals of the Rheumatic Diseases*.

[3] Roychoudhury A, Parkash H, Trikha A (1999). Functional restoration by gap arthroplasty in temporomandibular joint ankylosis a report of 50 cases. *Oral Surgery, Oral Medicine, Oral Pathology, Oral Radiology, and Endodontics*.

[4] Sidebottom AJ (2008). Guidelines for the replacement of temporomandibular joints in the United Kingdom. *British Journal of Oral and Maxillofacial Surgery*.

[5] Nayak PK, Nair SC, Krishnan DG, Perciaccante VJ, Ward Booth P, Schendel S, Hausamen JE (1999). Ankylosis of the temporomandibular joint. *Maxillofacial Surgery*.

[6] Abbe R (1880). An operation for the relief of anchylosis of the temporo-maxillary joint, by exsection of the neck of the condyle of the lower jaw; with remarks. *New York Medical Journal*.

[7] Punnia Moorthy A, Finch LD (1983). Interpositional arthroplasty for ankylosis of the temporomandibular joint. *Oral Surgery Oral Medicine and Oral Pathology*.

[8] Feinberg SE, Larsen PE (1989). The use of a pedicled temporalis muscle-pericranial flap for replacement of the TMJ disc: preliminary report. *Journal of Oral and Maxillofacial Surgery*.

[9] Erdem E, Alkan A (2001). The use of acrylic marbles for interposition arthroplasty in the treatment of temporomandibular joint ankylosis: follow-up of 47 cases. *International Journal of Oral and Maxillofacial Surgery*.

[10] Kent JN, Misiek DJ, Worthington P, Evans JR (1994). Controversies in disc and condyle replacement for partial and total temporomandibular joint reconstruction. *Controversies in Oral and Maxillofacial Surgery*.

[11] Cope MR, Moos KF, Hammersley N (1993). The compressible silicone rubber prosthesis in temporomandibular joint disease. *British Journal of Oral and Maxillofacial Surgery*.

[12] Saeed NR, McLeod NMH, Hensher R (2001). Temporomandibular joint replacement in rheumatoid-induced disease. *British Journal of Oral and Maxillofacial Surgery*.

[13] Ohara K, Nakamura K, Ohta E (1997). Chest wall deformities and thoracic scoliosis after costal cartilage graft harvesting. *Plastic and Reconstructive Surgery*.

[14] Mercuri LG, Edibam NR, Giobbie-Hurder A (2007). Fourteen-year follow-up of a patient-fitted total temporomandibular joint reconstruction system. *Journal of Oral and Maxillofacial Surgery*.

[15] Manemi RV, Fasanmade A, Revington PJ (2009). Bilateral ankylosis of the jaw treated with total alloplastic replacement using the TMJ concepts system in a patient with ankylosing spondylitis. *British Journal of Oral and Maxillofacial Surgery*.

[16] Agarwal A, Moll JMH (1980). Preankylosing spondylitis. *Ankylosing Spondylitis*.

[17] Chou CT, Pei L, Chang DM, Lee CF, Schumacher HR, Liang MH (1994). Prevalence of rheumatic diseases in Taiwan: a population study of urban, suburban, rural differences. *Journal of Rheumatology*.

[18] Crum RJ, Loiselle RJ (1971). Temporomandibular joint symptoms and ankylosing spondylitis. *The Journal of the American Dental Association*.

[19] Maes HJ, Dihlmann W (1968). Affection of the temporomandibular joints in spondylitis ankylopoeiticaBefall der Temporomandibulargelenke bei der Spondylitis ankylopoetica. *Fortschritte auf dem Gebiete der Rontgenstrahlen und der Nuklearmedizin*.

[20] Resnick D (1974). Temporomandibular joint involvement in ankylosing spondylitis. Comparison with rheumatoid arthritis and psoriasis. *Radiology*.

[21] Locher MC, Felder M, Sailer HF (1996). Involvement of the temporomandibular joints in ankylosing spondylitis (Bechterew’s disease). *Journal of Cranio-Maxillo-Facial Surgery*.

[22] Wolford LM, Pitta MC, Reiche-Fischel O, Franco PF (2003). TMJ concepts/techmedia custom-made TMJ total joint prosthesis: 5-year follow-up study. *International Journal of Oral and Maxillofacial Surgery*.

[23] Sidebottom AJ, Speculand B, Hensher R (2008). Foreign body response around total prosthetic metal-on-metal replacements of the temporomandibular joint in the UK. *British Journal of Oral and Maxillofacial Surgery*.

